# Converting Sugars to Biofuels: Ethanol and Beyond

**DOI:** 10.3390/bioengineering2040184

**Published:** 2015-10-27

**Authors:** Aram Kang, Taek Soon Lee

**Affiliations:** 1 Joint BioEnergy Institute, Emeryville, CA 94608, USA; 2 Biological Systems & Engineering Division, Lawrence Berkeley National Laboratory, Berkeley, CA 94720, USA; E-Mail: akang@lbl.gov

**Keywords:** biofuels, ethanol, advanced biofuels, lignocellulosic biomass, metabolic engineering

## Abstract

To date, the most significant sources of biofuels are starch- or sugarcane-based ethanol, which have been industrially produced in large quantities in the USA and Brazil, respectively. However, the ultimate goal of biofuel production is to produce fuels from lignocellulosic biomass-derived sugars with optimal fuel properties and compatibility with the existing fuel distribution infrastructure. To achieve this goal, metabolic pathways have been constructed to produce various fuel molecules that are categorized into fermentative alcohols (butanol and isobutanol), non-fermentative alcohols from 2-keto acid pathways, fatty acids-derived fuels and isoprenoid-derived fuels. This review will focus on current metabolic engineering efforts to improve the productivity and the yield of several key biofuel molecules. Strategies used in these metabolic engineering efforts can be summarized as follows: (1) identification of better enzymes; (2) flux control of intermediates and precursors; (3) elimination of competing pathways; (4) redox balance and cofactor regeneration; and (5) bypassing regulatory mechanisms. In addition to metabolic engineering approaches, host strains are optimized by improving sugar uptake and utilization, and increasing tolerance to toxic hydrolysates, metabolic intermediates and/or biofuel products.

## 1. Introduction: Sources of Sugars for Biofuel Production

**Figure 1:**
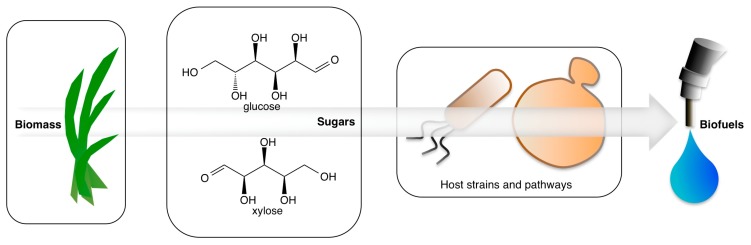
Overall scheme of biofuel production from biomass.

Ethanol and biodiesels have been industrially produced from biomass by fermentation and chemical trans-esterification of plant oils, respectively. For example, sugarcane-derived sugars (sucrose) have been used for ethanol fermentation in Brazil, and corn-derived starches (glucose) have been the major feedstock in the USA. Since consumption of these feedstocks for biofuel production competes with demands for animal feeds and human consumption [1], lignocellulosic biomass (LCB) has been suggested as an alternative and sustainable feedstock for biofuel industries. In using biomass for microbial fermentation, both non-LCB and LCB require pretreatment and hydrolysis of raw feedstock to release fermentable sugars from biomass consisting of complex and polymeric structures (Figure 1). The hydrolysis process of non-LCB such as corn starch has been well established in existing fermentation industries, but deconstruction of LCB has been limited due to the resistance of LCB against chemical and enzymatic treatment [1]. Moreover, hydrolysates of LCB include a mixture of pentose and hexose, inhibitory compounds (e.g., furfural, phenols) and toxic solvents produced during pretreatment, which all make downstream microbial fermentation difficult. Therefore, there have been studies to establish microbial hosts that co-utilize pentose and hexose, and to engineer tolerance of the microbial hosts against the above-mentioned toxic components [1].

Beyond native fermentation pathways or natural biodiesel resources such as vegetable oils, advanced biofuel molecules are now synthesized in microbial hosts where heterologous or synthetic metabolic pathways are reconstructed in fermentative hosts. In this review, we will summarize recent achievements and progress in microbial biofuel production with updates on biofuel production from LCB-derived sugars. Various pathways and hosts for ethanol and advanced biofuel production will be discussed, with a particular emphasis on metabolic engineering strategies to improve the microbial conversion bioprocess.

## 2. Fermentation Pathways and Hosts for Ethanol Production

**Figure 2:**
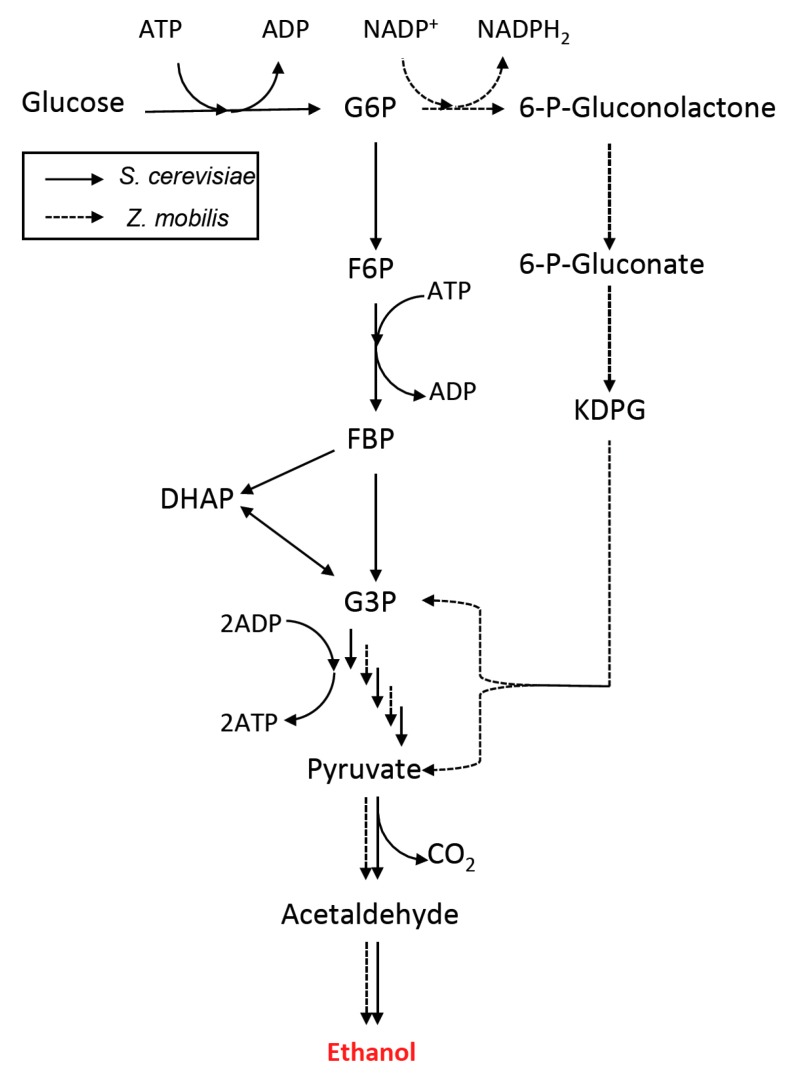
Ethanol fermentation pathways in *S. cerevisiae* (solid line, EMP glycolysis pathway) and *Z. mobilis* (dashed line, ED glycolysis pathway). While the EMP pathway produces two ATPs per glucose molecule, the ED pathway produces only one ATP molecule per glucose molecule. KDPG, 2-keto-3-deoxy-6-phosphogluconate; G6P, glucose 6-phosphate; F6P, fructose 6-phosphate; FBP, fructose 1,6-diphophate; DHAP, dihydroxyacetone phosphate; G3P, glyceraldehyde 3-phosphate.

Ethanol is produced from glucose via fermentative consumption of pyruvate [2]. Glycolysis is a metabolic process that converts glucose to partially oxidized product, pyruvate, while supplying ATP for biomass production. Subsequently, under anaerobic conditions, pyruvate can be fermented to ethanol by sequential reactions of pyruvate decarboxylase (PDC) and alcohol dehydrogenase (ADH) while losing one carbon as carbon dioxide (CO_2_). The ethanol fermentation process has been extensively studied and exploited in *Saccharomyces cerevisiae* (yeast) and *Escherichia coli* [1,3], due to the relative technological maturity in genetically engineering these microbes. Other species have also been considered as production hosts due to advantages of their native enzymes and pathways. For instance, *Zymommonas mobilis* has been suggested as an alternative host to yeast due to its advantage for ethanol yield since it utilizes the Entner-Doudoroff (ED) pathway instead of Embden-Meyerhof-Parnas (EMP) pathway for glycolysis [4] (Figure 2). Although the EMP pathway is a major glycolysis route in most eukaryotes and prokaryotes, glycolysis pathways are much more diverse in prokaryotes [5]. Among variants of the glycolysis pathway, the ED pathway is the most abundant route together with EMP pathway in some prokaryotes such as *Z. mobilis* [6]. While the EMP pathway produces two ATPs from each glucose molecule consumed, the ED pathway produces only one ATP molecule from one glucose molecule. Given that ATP is tightly coupled with anabolism and cell growth, ED pathway-utilizing *Z. mobilis* produces less biomass than EMP pathway-dependent species such as *S. cerevisiae* and *E. coli*. Consequently, *Z. mobilis* has more available carbons for ethanol fermentation with 2.5-fold higher specific ethanol productivity than that of *S. cerevisiae*, and produces up to 97% theoretical yield [7]. In addition, *Z. mobilis* has been engineered to co-utilize glucose, mannose and xylose, expanding their capability for ethanol fermentation of LCB-derived sugars [8]. Clostridia, on the other hand, have advantages over *S. cerevisiae* because Clostridia naturally secrete enzymes that are capable of hydrolyzing complex carbohydrates (oligosaccharides and polysaccharides) into fermentable sugars and utilizing both hexose and pentose [9,10]. As a result, Clostridia have been suggested as a candidate host for biofuel production from LCB for consolidated bioprocessing (CBP). However, Clostridia are strict anaerobes and their growth is relatively slower than other microbial hosts, which makes fermenter operation difficult. Various aspects of Clostridia for industrial use have been well summarized in a previous review [11].

In this section, we briefly introduce various aspects on selection of microbial hosts for ethanol production: energetics of the glycolysis pathways, available genetic engineering tools, flexibility in sugar utilization and compatibility to effective fermenter operation. The following section will discuss metabolic engineering strategies that have been applied to optimize and improve microbial hosts for ethanol production.

## 3. Metabolic Pathway and Host Engineering for Ethanol Production

Since sugars are both carbon and energy sources for biomass and ethanol production, more efficient uptake and utilization of various sugars are important factors that can improve ethanol productivity. For example, the uptake rate of sucrose was improved in yeast for more efficient utilization of sucrose from sugarcanes [12]. In this study, a *S. cerevisiae* strain with intracellularly localized sucrose invertase (iSUC1) was evolved in sucrose-limited chemostat, and the evolved strain showed higher sucrose-proton symporter activity and increased ethanol yield by 11% [12]. In general, most industrial microbial hosts have a good capability of utilizing hexose, but restricted capability in utilizing pentose such as xylose, the second most abundant sugar in biomass, and arabinose due to lack of the pentose utilization pathway and a catabolite repression in the presence of glucose. Although there have been efforts to expand the substrate utilization capability of yeast by engineering the substrate affinity of sugar transporters towards pentose rather than hexose, no significant progress has been made yet [13]. In one study, transporter mutants of GXS1 from *Candida intermedia* and XUT3 from *Scheffersomyces stipitis* were expressed, and it showed an increased growth rate of yeast on xylose by 70% and a changed pattern of diauxic shifts [14]. In a following study, Young and colleagues identified a sequence motif of G-G/F-XXX-G, of which saturation mutagenesis generated transporter mutants that have an exclusive specificity for xylose but not for glucose. All these mutants, however, were still found to be repressed by glucose [15]. In another study, Farwick and colleagues [16] screened glucose-insensitive xylose transporter mutants. In this study, they found a mutation at either of two conserved residues located near the entrance of sugar-binding pocket, and a mutant of a yeast hexose transporter (Gal2-N376F) was identified to have the highest affinity for xylose among mutants without glucose transport activity [16].

Another metabolic engineering strategy is to maximize the flux of sugars to ethanol while minimizing the flux to biomass or to other fermentation byproducts such as glycerol. Since formation of highly reduced fermentation products such as glycerol is driven by accumulation of cytosolic NADH, *S. cerevisiae* have been engineered to maintain lower level of cytosolic NADH by various genetic modifications such as the deletion of NADPH-dependent glutamate dehydrogenase (GDH1) along with the overexpression of glutamate-ammonia ligase (GLN1) and glutamate transporter (GLT1); and substitution of innate glyceraldehyde-3-phosphate dehydrogenase (GAPDH) with heterologous GAPDH from either *Bacillus cereus* or *Streptococcus mutans*. The latter approach aimed to decrease cytosolic NADH formation and ATP production by using an alternative non-phosphorylating, NADP^+^-dependent glyceraldehyde 3-phosphate dehydrogenase (GAPN) of GAPDH [13]. In addition to the resorts to decrease cytosolic NADH, metabolic pathways of ethanologenic *E. coli* were redesigned at the systems level by elementary mode analysis for metabolic coupling of biomass and ethanol production. Deletion of 9 genes in central metabolism was suggested by elementary mode analysis, and the engineered *E. coli* strain produced 90% of theoretical yield after 48 h fermentation [17]. On the other hand, carbon fluxes to biomass, organic acids and ethanol were re-distributed by heterologously expressing pyruvate decarboxylase (PDC) and alcohol dehydrogenase (ADHII) of *Z. mobilis* in *Streptomyces lividans* TK24 [18].

In addition to metabolic engineering efforts to diversify sugar utilization of microbial hosts and to regulate carbon metabolism fluxes, there have been other approaches to improve ethanol production, especially by improving the industrial bioprocess. One example is to improve resistance toward ethanol itself, growth inhibitors and toxic components from LCB-hydrolysates as well as other general stresses in ethanol-producing hosts [13]. Recently, extensive studies have been performed on ethanol production from LCB, and the advances are well summarized in a recent review [1]. Co-utilization of heterogeneous sugars in LCB hydrolysates still remains an unresolved issue in the production of LCB-derived ethanol. Since cellular processes involved in sugar consumption are complex, more systematic engineering of various factors such as transporters, regulatory mechanisms of catabolites and cellular responses to stress caused by intermediates and products would be required. Improved sugar utilization capability will benefit not only ethanol production from LCB but also the production of advanced biofuels from LCB, which will be discussed in the following section.

## 4. Metabolic Pathway and Host Engineering for Advanced Biofuels Production

Although ethanol is the most widely produced biofuel together with biodiesels, ethanol is not an ideal alternative fuel or blending fuel due to its low energy content (only about 70% of gasoline) and hygroscopic nature [19]. As a result, there has been significant demand for advanced “drop-in” biofuels that have better fuel properties and are compatible with the current engines and infrastructure. Good alternative transportation fuels would have similar chemical structures and properties to those found in existing transportation fuels (gasoline, diesel, and jet fuels). Metabolic pathways that produce desirable fuel-like molecules have been engineered: fermentative alcohols (butanol and isobutanol), non-fermentative alcohols from 2-keto acid pathways, fatty acids-derived fuels and isoprenoid-derived fuels.

**Figure 3:**
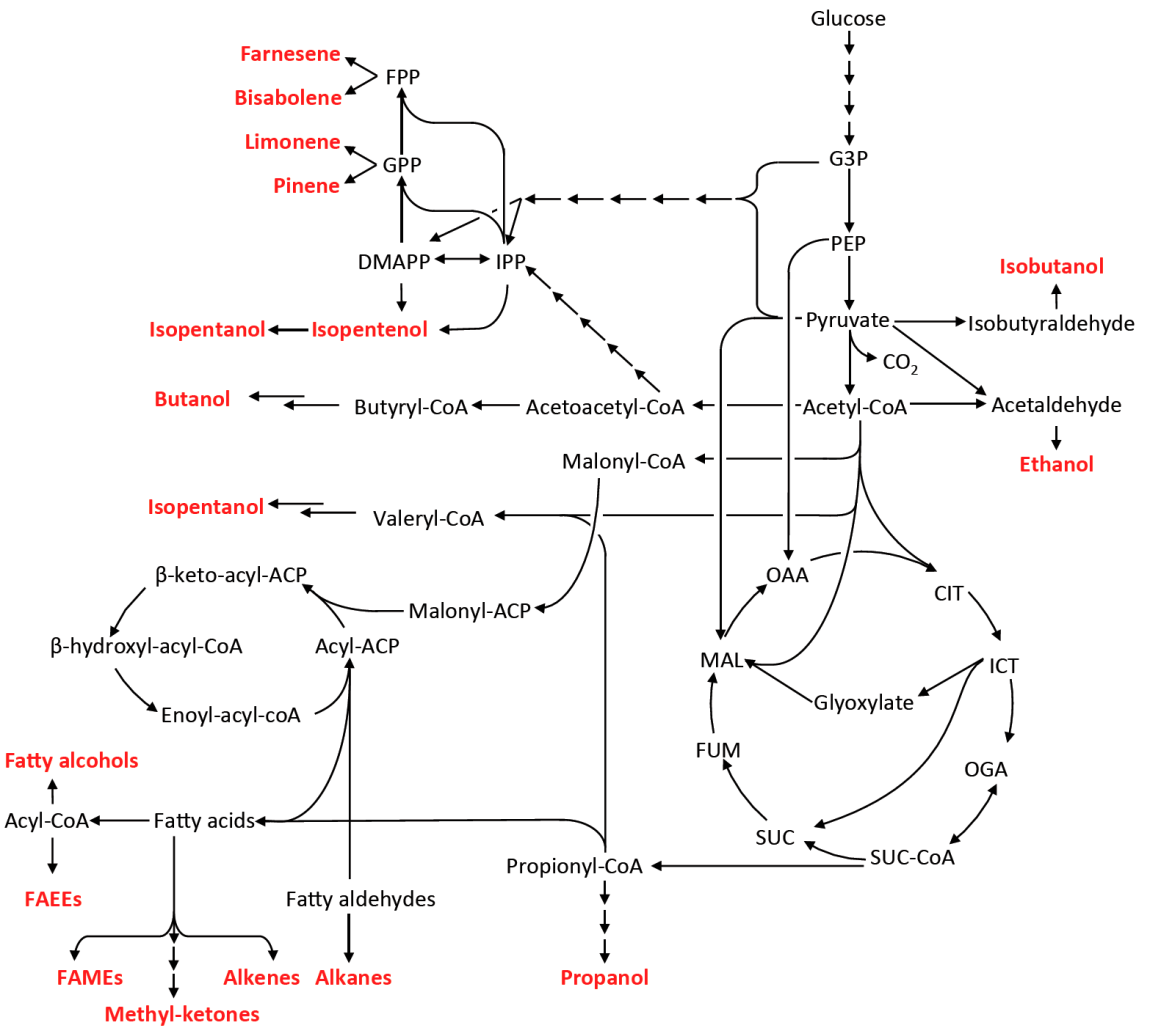
Metabolic pathways to advanced biofuel molecules. ACP, acyl carrier protein; IPP, isopentenyl diphosphate, DMAPP, dimethylallyl diphosphate; FPP, farnesyl diphosphate; GPP, geranyl diphosphate; G3P, Glyceraldehyde 3-phosphate; PEP, phosphoenolpyruvate; CIT, citrate; ICT, isocitrate; OGA, oxoglutarate; SUC-CoA, succinyl-CoA; SUC, succinate; FUM, fumarate; MAL, malate; OAA, oxaloacetate; FAEEs, fatty acids ethyl esters; FAMEs, fatty acid methyl esters.

The general overview of the pathways for advanced biofuel production is summarized in Figure 3 and Table 1.

**Table 1:** Production titer of various advanced biofuel molecules and theoretical yields.

Product	Pathway	% of Apparent Theoretical Yield (from Glucose)	Highest Titer Reported (g/L)	Host	Reference
**Gasoline**					
1-Butanol	CoA-dependent	41% ^a^	30	*E. coli*	[20]
Isobutanol	2-keto acids	41% ^a^	50	*E. coli*	[21]
3-methyl-1-butanol	2-keto acids	33% ^a^	9.8	*E. coli*	[22]
3-methyl-3-butenol	MVA	41% ^b^	2.2	*E. coli*	[23]
2-methyl-1-butanol	2-keto acids	39% ^b^	1.25	*E. coli*	[24]
**Diesel & Jet Fuel**					
Farnesene	MVA	25% ^a^	NA		[25]
Farnesene	MEP	29% ^a^	NA		[25]
Bisabolene	MVA	25%	1.15	*E. coli*	[26]
Limonene	MVA	25%	0.605	*E. coli*	[26]
Pinene	MVA	25%	0.032	*E. coli*	[27]
FAEE	Fatty acids	35% ^a^	1.5	*E. coli*	[28]
Methyl ketones	Fatty acids	33%	3.4	*E. coli*	[29]

**^a^** Rude and Schirmer, 2009 [25]; **^b^** Dugar and Stephanopoulos, 2011 [30].

### 4.1. Fermentative Pathways for 1-Butanol and Other Short Chain Alcohols

1-butanol is naturally produced by species of Clostridia, which have innate 1-butanol fermentative pathways. The 1-butanol fermentation pathway and general aspects of Clostridia physiology (e.g., sporulation cycle and acidogenesis) were summarized along with metabolic engineering efforts to improve 1-butanol fermentation in the recent review [9]. Other specific aspects regarding 1-butanol fermentation by Clostridia have also been reviewed with a particular focus on the diversity of Clostridia strains for ABE (Acetone-Butanol-Ethanol) fermentation and 1-butanol fermentation on various substrates [31]. Metabolic engineering strategies to overcome limitations in Clostridia butanol fermentation are (i) redox balancing (e.g., regeneration of NADH via butanol fermentation, which could be increased by reducing hydrogenase activity [9]); (ii) reducing byproduct formation and improving 1-butanol productivity; and (iii) conferring host tolerance against 1-butanol [32].

Although Clostridia species are natural 1-butanol producers, they grow slowly and their genetic manipulation is still limited. To overcome these limitations, the 1-butanol fermentation pathway was re-constructed in *E. coli* by incorporating seven enzymes from three different species [33]. One of the key engineering strategies was substituting a flavin-dependent native Bcd/EtfAB system of Clostridia to an irreversible enoyl-CoA reductase (Ter) from *Treponema denticola* [34]. This Ter-based, synthetic pathway has been further improved by building up NADH and acetyl-CoA as a driving force for 1-butanol production under anaerobic conditions. Accumulation of NADH and acetyl-CoA could be achieved by eliminating four fermentation pathways and by expressing formate dehydrogenase (*fdh1*), and as a result, the titer could reach up to 30 g/L in *E. coli* [20]. Another study optimized transcription level of *fdh1* to minimize redox imbalance by excessive regeneration of NADH, and it subsequently showed increased 1-butanol productivity in *E. coli* [35]. Furthermore, expression of *acrB* efflux pump conferred tolerance of *E. coli* to 1-butanol [36]. Not only 1-butanol, but isopropanol can be produced via fermentation by reducing acetone, one of the three fermentation products in Clostridium. The highest titer of up to 143 g/L was achieved with gas stripping via fermentation pathway [37].

### 4.2. Non-Fermentative Pathways for Short Chain Alcohols: 2-Keto Acid Pathway

**Figure 4:**
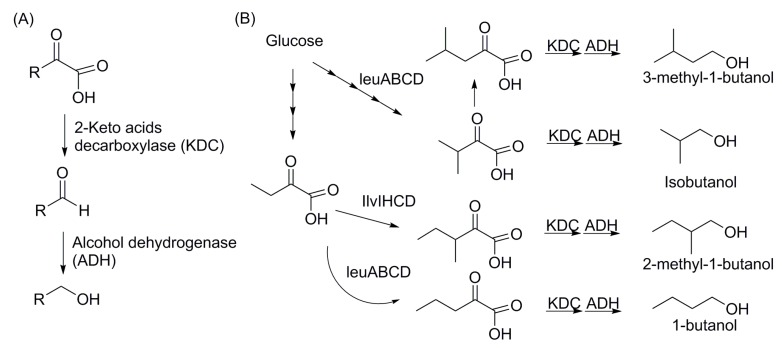
Non-fermentative pathways for short chain alcohols: 2-keto acid pathway. (**A**) General reaction from 2-keto acids to alcohols. 2-keto acid decarboxylase (KDC) catalyzes the decarboxylation of 2-keto acid to form the aldehyde. Then, alcohol dehydrogenase (ADH) reduces the aldehyde to the alcohol. (**B**) Examples of short chain alcohols synthesized from 2-keto acid pathway.

Short-chain alcohols can be produced via non-fermentative pathways such as the 2-keto acid pathways, and the latest advances in production titer and engineering approaches can be found in recent review papers [38,39]. In these pathways, 2-keto-acid intermediates are transformed to corresponding aldehydes and subsequently to alcohols by decarboxylases and alcohol dehydrogenases, respectively [40] (Figure 4). 1-butanol and branched-chain alcohols such as isobutanol (C_4_) and isopentanols (C_5_) are produced from keto acids intermediates of valine and leucine biosynthesis pathways by increasing availability of specific keto acids and by expression of promiscuous keto acid decarboxylase (KivD) from *Lactococcus lacti*s with alcohol dehydrogenase 2 (Adh2) from *S. cerevisiae* [38]. Subsequently, the highest isobutanol production titer from keto acid pathways has been achieved up to 50 g/L in *E. coli* with gas stripping [21]. Linear alcohols ranging from 1-pentanol (C_5_) to 1-octanol (C_8_) were also produced from threonine over-producing *E. coli* [41]. In this paper, the carbon chain of 2-keto acids was recursively elongated by an engineered leucine synthesis pathway from *E. coli* (*Ec*LeuABCD). The production titer was about 1.4 g/L [41].

Alternative hosts such as amino acid overproducing *Corynebacterium glutamicum* and more isobutanol-tolerant *B. subtilis* have been also used, and mitochondrial targeting and expression of cytoplasmic Ehrlich pathway enzymes improved isobutanol production by 2.6-fold in yeast [42]. Recently, Tseng and colleagues demonstrated an alternative pathway to produce odd-carbon chemicals such as pentanol more efficiently with higher theoretical yield in *E. coli* by assembling different pathways modularly [43].

Short-chain alcohols have been produced via various metabolic pathways and to relatively higher titers than other advanced biofuel molecules. Even though they are considered to have better fuel properties than ethanol, there has still been an increasing demand for biofuel molecules with better properties such as higher energy content and lower freezing points. To address this issue, microbial hosts have been engineered to produce biofuel molecules with longer hydrocarbon chains and more branching methyl groups via various metabolic pathways such as fatty acid metabolic pathways (Section 4.3.) and isoprenoid pathways (Section 4.4.).

### 4.3. Fatty Acid-Based Biofuels

**Figure 5:**
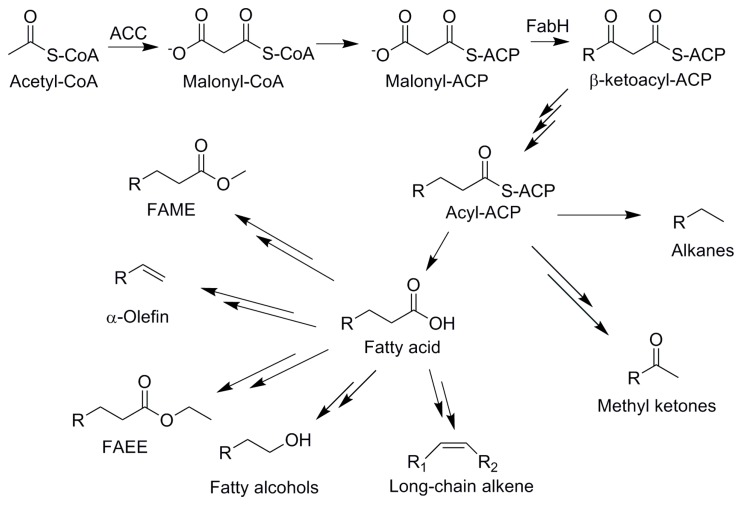
Overview of biofuel compounds derived from fatty acids pathways. ACC: acetyl-CoA carboxylase.

The energy-rich hydrocarbon chains of fatty acids make them potential precursors for the production of diesel alternatives. Fatty acids are synthesized by fatty acid synthase (FAS) system, which condenses malonyl-CoAs into various lengths of fatty acyl esters with acyl-carrier protein (ACP) (Figure 5). Currently, plant oils and animal fats are chemically converted to fatty acid alkyl esters (fatty acids ethyl esters, FAEEs and fatty acids methyl esters, FAMEs) via trans-esterification. Microbial production of FAEEs was facilitated by identification of promiscuous wax ester synthase/acyl-CoA:diacylglycerol acyltransferase (WS/DGAT), which was first characterized in *Acinetobacter calcoaceticus* ADP1 [44], and 1.28 g/L FAEE was produced with oleic acids feeding in *E. coli* strain where pyruvate decarboxylase (Pdc) and alcohol dehydrogenase (AdhB) from *Z. mobilis* were co-expressed to provide ethanol [45]. Conversion of sugars to FAEEs, fatty acid alcohols and wax esters without extracellular feeding of fatty acids or ethanol was demonstrated by expression of *pdc, adhB, tesA’* (membrane targeting sequence truncated *E. coli* native thioesterase) and f*adD* (native acyl-CoA synthetase) in *E. coli* with *fadE* gene deletion (*ΔfadE*), where fatty acid metabolism was forced to produce more fatty acyl-CoA [46], an important precursor for various fatty acid-derived fuels. The introduction of a dynamic sensor-regulator system significantly increased fatty acid production several times higher in *E. coli* by balancing substrate supply levels [28], which resulted in a FAEE titer of 1.5 g/L with 28% of maximum theoretical yield (Table 1).

Fatty acid-derived fuels were also produced in yeast by overexpressing all three fatty acid biosynthesis genes (ACC1, FAS1 and FAS2) in combination with the expression of downstream enzymes (diacylglycerol acyltransferase, fatty acyl-CoA thioesterase, fatty acyl-CoA reductase, and wax ester synthase) [47]. Oleaginous yeast species are now extensively studied as promising hosts for FAEE production since they naturally produce and accumulate a large amount of lipids—up to 36% of their dry weights. Recent studies increased the intracellular lipid content even further, up to 40%–70%, by engineering *ex novo* lipids biosynthesis, and it has been shown that lipids could be accumulated via tightly regulated *de novo* synthesis pathway when acetyl-CoA carboxylase (ACC1) and diacylglycerol acyltransferase (DGA1) were overexpressed [48].

Other fatty-acid-derived biofuel molecules such as fatty aldehydes, methyl ketones, alkanes and alkenes are now produced in microbial hosts (Figure 5), and production of those fatty acid-derived fuels were reviewed recently [49,50]. The most significant progress has been made by identifying biochemical pathways and responsible enzymes to produce targeted fuel molecules from various organisms and by reconstruction of the heterologous pathway in selected hosts. For example, heterologous expression of acyl-ACP reductase and aldehyde deformylase [51] (previously known as a fatty aldehyde decarbonylase) enabled *E. coli* to produce alkanes and alkenes [52]. Furthermore, two terminal alkene synthesis enzymes were discovered for production of α-olefins; elongase decarboxylase (a homologous enzyme to type I polyketide synthase) from *Synechococcus sp.* PCC7002 [53] and fatty acid decarboxylase (a cytochrome P450, OleT_JE_) from *Jeotgalicoccus* species [54], which act on acyl-ACP and fatty acids, respectively. In addition to understanding the biochemistry and structure of the identified enzymes, finding alternative enzymes to reduce fatty acids pathway intermediates to fuel molecules have been of great interest to the research community. For example, NADPH-dependent fatty aldehyde reductase from *Marinobacter aquaeolei* VT8 exhibited reducing activity for both acyl-CoAs and subsequently produced aldehydes [55], which suggests that expression of this enzyme possibly reduces accumulation of toxic aldehyde intermediates [49]. Lastly, the methyl ketone pathway has been engineered in an *E. coli* strain with *fadA* gene deletion by overexpression of β-ketoacyl-ACP synthase II (FadB), an acyl-CoA oxidase from *Micrococcus luteus*, and a thioesterase (FadM) to produce β-keto fatty acids followed by hydrolysis and decarboxylation to ketones [29,56].

Since fatty acids are the primary precursors, many engineering approaches have focused on increasing available fatty acids [39]. Those approaches include overexpression of thioesterases, blocking fatty acids degradation via β-oxidation, genomic modification to increase metabolic flux to malonyl-CoAs and balancing FA pathway intermediates by sensor-regulator and FA synthase subunits. In addition, novel and synthetic pathways for fatty acid production were proposed. Most significantly, fatty acids were over-produced by reversing β-oxidation while avoiding ATP consumption and tight regulation related to acetyl-CoA carboxylase, which produces the FA synthesis precursor, malonyl-CoA [57]. Using this synthetic pathway, fatty acids were produced up to ~7 g/L (up to 80% of its theoretical maximum yield) [57]. In another study, the carbon chain lengths of fatty-acid-derived fuels were extended in the range from C_6_ to C_18_ in *E. coli* by introducing alternative carboxylic acids reductase from *Mycobacterium marinum* [58]. Furthermore, it was reported that the composition of fatty acid-derived biofuels could mimic that of diesel or aviation fuel when free fatty acid pools were modified to have iso-branched fatty acids and alkanes in *E. coli* [59]. Composition of iso-branched fatty acids could be further increased to 20% of the total fatty acids by expression of biosynthesis genes for threonine and isoleucine, although the total titer of fatty acids was decreased [60].

### 4.4. Isoprenoid-Based Biofuels

**Figure 6:**
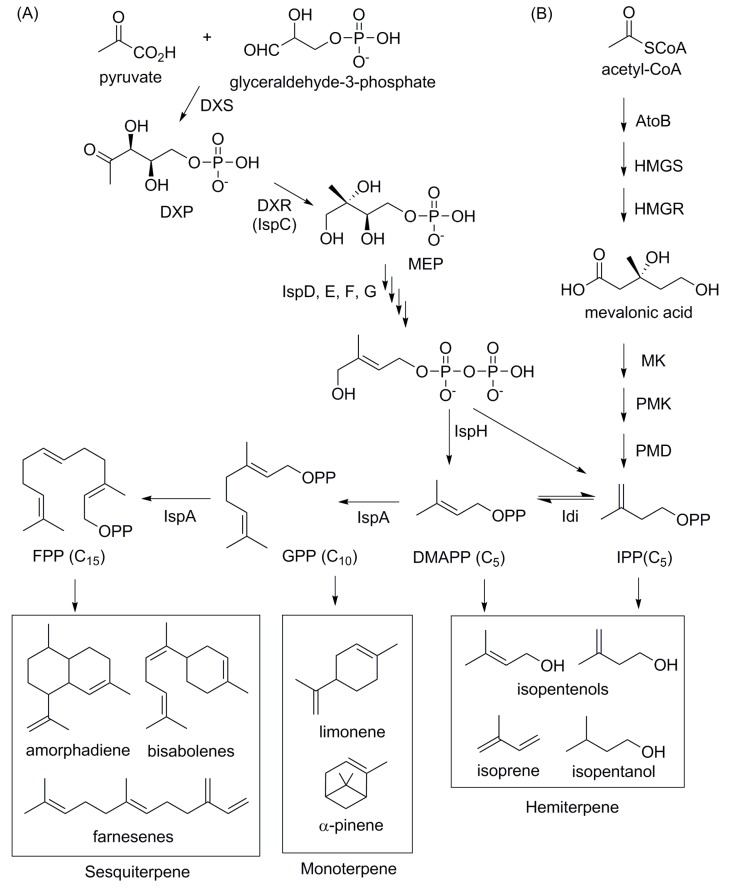
Isoprenoid pathways ((A) MEP and (B) MVA pathways) and biofuel compounds derived from isoprenoid pathways. DXS: deoxyxylulose-5-phosphate (DXP) synthase, MEP: methylerythritol phosphate, DXR: DXP reductase, HMGS: 3-hydroxy-3-methylglutaryl-coenzyme A (HMG-CoA) synthase, HMGR: HMG-CoA reductase, MK: Mevalonate kinase, PMK: Phosphomevalonate kinase, PMD: Phosphomevalonate decarboxylase.

Isoprenoids are a group of diverse chemical compounds which includes over 50,000 compounds. Fuel molecules derived from isoprenoid pathways have branched hydrocarbon chains, which lower their freezing temperature, as well as various ring structures, which makes them potential alternatives to diesel and jet fuels [61]. These branched hydrocarbon chains are derived from two universal precursors, isopentenyl diphosphate (IPP) and its isomer, dimethylallyl diphosphate (DMAPP) (Figure 6). The isoprene unit of IPP is first condensed to DMAPP and iteratively further condensed to various length of prenyl diphosphate molecules such as geranyl diphosphate (C_10_, GPP), farnesyl diphosphate (C_15_, FPP) and geranylgeranyl diphosphate (C_20_, GGPP) [62]. These condensation reactions are catalyzed by prenyltransferase enzymes, of which specificity is the determining factor of hydrocarbon length of the relevant isoprenoid [63]. Finally, prenyl diphosphates are diversified to various structures of isoprenoids by terpene synthase enzymes, mostly via carbocation formation [64]. Regardless of the final structures, isoprenoids are primarily classified into different groups based on the length of the hydrocarbon backbone, and the most abundant isoprenoids belong to monoterpenes (C_10_), sesquiterpenes (C_15_) and diterpenes (C_20_). Therefore, engineering efforts to produce isoprenoids-derived biofuels have been focusing on increasing production of prenyl diphosphate intermediates, particularly IPP and DMAPP, and identification and engineering of terpene synthases for desired enzymatic activity.

Although two different biosynthetic pathways (the mevalonate (MVA) pathway and methylerythritol phosphate (MEP) pathway) have been known to produce two universal C_5_ precursors, the MVA pathway has been more extensively exploited for production of potential diesel and jet fuel precursors such as farnesene [65], bisabolene [66,67], pinene [27,68] and limonene [26,69] (Figure 6). Since isoprenoid-derived fuels are dependent on the two precursors, IPP and DMAPP, most engineering efforts are focused on optimizing pathways by balancing fluxes of metabolic intermediates, increasing the transcription level of limiting enzymes [70], improving protein expression by codon-optimization, and reducing reversibility of the reactions [71].

Among sesquiterpene-derived fuels, biological production of farnesene and bisabolene has been reported. Farnesene is converted from FPP by farnesene synthase. The previously developed FPP-overproducing *E. coli* and yeast strains led to an initial farnesene titer of 1.1 g/L after 120 h of *E. coli* fermentation and 728 mg/L after 72 h of yeast fermentation [72,73]. After continuous evolution of the host strains, Amyris, a biotech company, reported farnesene production to the titer of 104.3 g/L from the engineered yeast strain [74] and commercialized trans-β-farnesene under the name, “Biofene®”. Bisabolenes are another group of sesquiterpenes, of which fuel properties are qualified for diesel alternatives [67]. Initially, a titer of 400–800 mg/L of bisabolene was produced from *E. coli* and yeast platforms engineered for FPP-overproduction [70,75]. About 40% increase in titer was reported in *E. coli* by applying principal component analysis of proteomics (PCAP) approach [26]. In yeast, three genes that encode two unknown functions and a transcriptional regulator, Rox1, were identified from carotenoid-based screening method, and further engineering efforts resulted in 5.2 g/L bisabolene titer in fed-batch fermentation conditions [66].

Microbial production of monoterpenes was significantly improved by increasing availability of GPP and protein expression level of terpene synthases. For example, production of limonene, a promising diesel fuel precursor [76], has been significantly improved by introducing a heterologous MVA pathway and optimizing GPP synthase from *Mentha spicata* and limonene synthase from *Abies grandis* [69]. A step-by-step optimization led to over 100-fold titer increase from the initially reported titer using the MEP pathway [77] up to 450 mg/L and further engineering using proteomics analysis of the pathway enzymes improved the titer about 40% [26]. For a jet-fuel precursor pinene production, initial titer in *E. coli* (5.44 mg/L in flasks [78]) has been significantly improved by combinatorial fusion of GPP synthase and pinene synthase, reporting the highest titer of 32 mg/L when GPP synthase and pinene synthase from *A. grandis* were co-expressed as a fused protein [27]. In addition to limonene and pinene, microbial production of sabinene was also reported with the titer of 82.18 mg/L [79].

Production of isoprenoid-derived alcohols were achieved by co-expression of phosphatases: isopentenol [23,80,81,82], geraniol [83] and farnesol [73]. Particularly, isopentenol, a promising biofuel and a precursor for commodity chemicals such as isoprene, was produced at a titer of 2.2 g/L from 10 g/L glucose (70% of apparent theoretical yield) [23]. This significant improvement in isopentenol production has been achieved by the “fine-tuning” of the upstream MVA pathway [81] and the increased availability of NudB, which is required for hydrolysis of IPP into isopentenol [23].

### 4.5. Advanced Biofuels Production from LCB-Derived Sugars or Hydrolysates

Biofuel production from LCB has been primarily limited by pretreatment and saccharification of LCB, which determines sugar yields, and by inefficient co-utilization of hexose and pentose of host strains. While ethanol production has been more frequently chosen as a representative pathway to demonstrate biofuel production from LCB-derived sugars, a few studies on advanced biofuels production from LCB-derived sugars have been pursued recently [84]. One of the early studies demonstrated the engineering of *E. coli* as a microbial factory for consolidated bioprocesses (CBP) by expressing enzymes required for both biomass degradation (cellulase, xylanase, β-glucosidase, and xylobiosidase) and biofuel synthesis (FAEE, butanol and pinene) [68]. Using this engineered *E. coli* strain, 71 mg/L of FAEE, 28 mg/L butanol, and 1.7 mg/L pinene were produced from 5.5%, 3.3% and 3.9% *w*/*v* IL-treated switchgrass, respectively. Although these titers need to be further improved for industrial application, it should be noted that 71 mg/L of FAEE was 80% of the estimated yield from 5.5% switchgrass that could release only 0.14% glucose and 0.14% xylose by the cellulose and the xylanase expressed from the engineered *E. coli* strain [68]. A recent study demonstrated simultaneous isopentenol fermentation and saccharification of ionic liquid (IL)-pretreated pellets containing a mixture of four feedstocks [85]. The IL-pretreated pellet released 7 g/L glucose in 48 h, and almost 1 g/L of isopentenol was produced out of this hydrolysate. In another study, *E. coli* was engineered to produce isobutanol from xylose, by integrating the isobutanol synthetic pathway and xylose utilization genes into the genome and using xylose as an inducer for the expression of these genes [86]. In this study, a titer of 3.6 g/L isobutanol was produced from cedar hydrolysates containing 86.4 g/L glucose and 15.5 g/L xylose although the productivity was 4.5 times lower than media containing pure glucose and xylose [86]. In addition, higher alcohols have been produced in *Corynebacterium crenatum* via keto acid pathways using acid-pretreated hydrolysates of duckweed [87]. In this work, heterologous genes involved in isoleucine, leucine and valine biosynthesis pathways from *S. cerevisiae* were expressed in *C. creanatum*, and 982 mg/L of 2-methyl-1-butanol, ~1.1 g/L isobutanol and ~685 mg/L of 3-methyl-1-butanol were produced from acid-pretreated hydrolysates of duckweed containing 60 g/L glucose without compromising productivity [87]. Although raw hydrolysates were not used as a carbon source, Avicel hydrolysates containing cellobionic acid, one of the major components of lignocellulosic biomass, were also used to produce 1.4 g/L of isobutanol achieving 36% of the theoretical maximum with a productivity of 0.03 g/L/h by expressing a native gene, *ascB* encoding 6-phospho-β-glucosidase [88]. Even though most studies showed that productivity was relatively reduced when biofuels were produced from LCB-derived hydrolysates or sugars, these results suggested that production of advanced biofuels from LCB-derived sugars is currently feasible, and it could be further improved by overcoming limitations that are not intrinsic to the engineered biofuel pathways and by further optimization of the responsible metabolic pathways.

## 5. Conclusions

Ethanol has been produced from various carbon sources (from corn- and sugarcane-based glucose to lignocellulosic biomass) by engineering or by exploiting native fermentation pathways of various microbial hosts. Production of higher alcohols (1-butanol, isobutanol, isopentanol, *etc.*) as alternatives to ethanol with better fuel properties has been demonstrated by engineering fermentative pathways, non-fermentative keto-acid pathways, and isoprenoid pathways. In addition to higher alcohols, fatty-acid-derived biofuels and isoprenoids-derived biofuels have also been proposed as good diesel alternatives. Various microbial hosts and metabolic pathways were explored extensively to improve yield, titer, and productivity using various strategies. It would be hard to establish a common strategy that works for all kinds of biofuels derived from various metabolic pathways. However, more systemic and more collective efforts would be required in the future to overcome several bottlenecks mentioned in this review, such as extended sugar utilization capability, robustness of microbial hosts against general stresses and toxic products, and scale-up and actual commercialization of advanced biofuels. These bottlenecks are related to the general physiology of microbial hosts rather than to any specific metabolic pathways. Metabolic pathway engineering in addition to improving general physiology of candidate biofuel producers would allow more economically viable biofuel production, which will reduce the heavy dependence on petroleum-based fuel and contribute to slowing down global warming by providing carbon-neutral energy for the transportation sector.
